# Mental Health, Mucosal Immunity, and HIV Susceptibility Following Sexual Violence: Evidence from the THRIVE Study

**DOI:** 10.3390/v18010119

**Published:** 2026-01-15

**Authors:** Katherine M. Anderson, Eleanor Capozzi, Stephanie A. Meyers-Pantele, Maile Y. Karris, Fernando Cabezas Mejia, Ella Meyer, Melodie A. Nasr, Mimi Ghosh, Jamila K. Stockman

**Affiliations:** 1 Department of Behavioral, Social, and Health Education Sciences, Rollins School of Public Health, Emory University, Atlanta, GA 30322, USA; kmande9@emory.edu; 2 Division of Infectious Diseases and Global Public Health, Department of Medicine, University of California, La Jolla, CA 92093, USA; s3meyers@health.ucsd.edu (S.A.M.-P.); m1young@health.ucsd.edu (M.Y.K.); jstockman@health.ucsd.edu (J.K.S.); 3 Department of Epidemiology, Milken Institute School of Public Health, The George Washington University, Washington, DC 20052, USA

**Keywords:** sexual violence, HIV prevention, mucosal immunity, cisgender women, rape, inflammation, mental health

## Abstract

Sexual violence against women is a global issue with profound health consequences, including elevated HIV risk due to genital tract inflammation and injury. However, limited research has examined the influence of mental health on HIV-related immunity after violence. We analyzed longitudinal data from female survivors of past-month rape (N = 25) to explore associations between mental health (perceived stress, depression, post-traumatic stress disorder [PTSD], and resilience) and HIV-associated immune biomarkers in the female genital tract. In bivariate analyses, mental health improved over the three-month follow-up period. Immune biomarker levels remained largely stable, except for TNF-α and SLPI. At baseline, depression was significantly correlated with TNF-α, IL-6, and IL-1β. In regression analyses, depression was associated with TNF-α (β = −0.133 to −0.152) and IL-6 (β = −0.171 to −0.207). PTSD was significantly associated with IL-1α (β = 0.576 to 1.681). Depression and resilience were negatively associated with percent HIV inhibition in adjusted models. These findings suggest that depression and PTSD are associated with genital tract inflammation following sexual violence, which may compromise mucosal immunity and enhance HIV risk. This highlights the importance of integrated mental health and immunological care for survivors and the need for further research into psychoneuroimmune pathways influencing HIV risk after trauma.

## 1. Introduction

Almost one-third of women globally experience intimate partner sexual violence in their lifetime [1], while almost 10% experience non-partner sexual violence [2]. These experiences include forced vaginal penetration and unwanted vaginal penetration after threats or coercion, otherwise known as rape, and are most often perpetrated by men [3]. Research has documented severe psychological, physiological, and social impacts of rape among survivors in the weeks, months, and years following these experiences [4,5,6,7,8,9]. Sexual violence is associated with numerous adverse psychological outcomes, including trauma-related conditions (e.g., post-traumatic stress disorder [PTSD], suicidality, depression, and anxiety) [6,7]. In the only study that prospectively examined the long-term mental health burden over a two-year post-rape period, 45.2% of the women met the cutoff for depression and 32.7% for PTSD, which was significantly higher than levels found among women not exposed to rape (33.1% for depression and 12.8% for PTSD) [8]. Additionally, some studies suggest that women who experience sexual violence have lower mental health-related quality of life and life satisfaction [9]. In a nationally representative sample of U.S. female survivors of rape, over 70% reported some physical health impact, with approximately 40% reporting injury from the assault itself [4]. Overall, survivors have significantly higher lifetime odds of many negative health outcomes, such as asthma, chronic pain, and difficulty in daily functioning [4,5]. While less commonly discussed, among these outcomes is the increased risk for HIV acquisition [10,11,12,13]. A 2013 systematic review of 101 articles identified that women who experienced intimate partner violence (IPV) had HIV prevalence 1.8 to 8 times greater than those without experiences of IPV; of the 92 studies with information on IPV subtype available, 75% included sexual IPV [12]. In a cohort study in South Africa, women who had experienced rape faced a 60% higher likelihood of HIV acquisition compared to controls, even when controlling for confounders [14]. Further, a 2025 meta-analysis identified that sexual violence increased HIV risk by at least 15%, with an average risk ratio indicating an 85% increased risk [15].

Two factors are thought to contribute to this increased risk for HIV: behavior and immunological dysfunction. Survivors of violence may engage in sexual- and drug-related HIV/STI risk behavior, including condomless sex and concurrent sex partners, responsive to violence-associated adverse mental health [14]. In one study, survivors of sexual violence had increased odds of drug use and sexual violence revictimization [9], both potential HIV transmission pathways. Such behaviors may lead to increased likelihood of HIV exposure. Compounding this, survivors of rape have been found to exhibit local and systemic immune dysregulation, potentially limiting the body’s ability to prevent HIV transmission upon exposure. In studies of peripheral immune biomarkers, immune dysregulation in the form of either chronic heightened immune activation (inflammation) or immune suppression [10,16] is associated with trauma, PTSD, and depression [17,18,19], manifesting in heightened or below-standard levels of interleukin (IL)-1β, IL-6, tumor necrosis factor (TNF)-α, IL-2, interferon (IFN)-γ, IL-10, and C-Reactive Protein (CRP) [18,19,20,21]. After rape, there is the added concern of mucosal trauma, which may lead to altered immune function and aberrant inflammatory responses [22], increasing susceptibility to sexually transmitted infections, including HIV [23]. In the few cross-sectional studies of women who have experienced sexual violence, researchers have identified higher levels of pro-inflammatory IL-1α and lower levels of monocyte chemoattractant protein-1 (MCP-1) in cervical fluid of survivors, compared to controls, while varying relationships have been identified with MIP-3α [22,24]. Separately, increased levels of pro-inflammatory cytokines (IL-1α, IL-1β, IL-6, TNF-α, IL-8, IFN-γ), inducible protein-10 (IP-10), monocyte chemoattractant protein-1 (MCP-1), and macrophage inflammatory protein (MIP-1α, MIP-1β) are also associated with HIV seroconversion [25], underscoring the complex risk profile associated with post-sexual violence incident.

Despite significant amounts of research on the impacts of violence against women, few studies have engaged survivors of sexual violence to understand the mechanisms responsible for immunological changes that lead to enhanced HIV risk. A recent case study followed a survivor of repeat sexual violence victimization and demonstrated co-variation in mental health indicators and immune biomarkers over time, underscoring the potential interrelation of these variables and their responsivity to events of sexual violence [26]. Multiple studies have explored the relationship between adverse mental health and immune biomarkers, some reporting immune activation while others finding immune suppression [17,27,28]. Primary analysis of data from this study [29] indicated higher perceived stress, depression, PTSD symptoms, TNF-α, and SLPI among women who had experienced recent sexual violence compared to controls, while moderation analysis identified that mental health differentially influenced IL1-α, IL-6, and IL-8 between groups. To our knowledge, little research beyond this has assessed sexual violence, immune functioning, and HIV/STI risk together, or tracked these outcomes over time following sexual violence. As such, our objective was to understand changes in mental health indicators (perceived stress, depression, PTSD, resilience) and HIV-associated immune biomarkers in the female genital tract (FGT) in the three months immediately following experiences of sexual violence among case participants enrolled in the study. We hypothesized that, based on the available literature and preliminary data, mental health indicators and immune biomarkers would demonstrate changes in the months after sexual violence, and that at each timepoint, immune biomarkers would be correlated with mental health indicators.

## 2. Materials and Methods

### 2.1. Study Procedures

The THRIVE Study was a case–control study of women aged 14 to 45 living in California, who had experienced recent forced vaginal penetration (cases) or recent consensual vaginal penetration (controls). Eligible participants were at least 14 years old, were required to have a vagina, cervix, and uterus, be HIV-negative upon enrollment, and have experienced vaginal penetration perpetrated by a man, consensually or forced, by a phallus within the past 15 days. Due to difficulties in enrollment, the timeframe for enrollment after sexual violence was expanded for case participants to one month. Exclusion criteria included current pregnancy or breastfeeding, use of vaginal cleansing products within the past 7 days, or cognitive impairment limiting ability to consent/assent and participate in the study. Additionally, for cases, exclusion criteria included having had consensual penetrative vaginal sex between sexual violence exposure and enrollment in the study. In total, The THRIVE Study enrolled 68 women, including 25 case participants and 43 control participants. The present analysis includes the sub-sample of case participants only (*n* = 25); no control participant data was analyzed in the current analysis. Details of this study are available in-depth elsewhere [29], as are results of differences between case and control participants [30]; details pertinent to the current analysis are outlined below.

Participants were recruited through social media advertising, referral from community sources including a rape crisis center, and distribution of flyers. Potential participants completed either a 5 min online screener survey on REDCap hosted by the University of California San Diego Altman Clinical and Translational Research Institute, or a phone-based eligibility screener with a trained staff member. Participants aged 18 and older provided written informed consent. While the study was open to adolescents aged 14 and over, no participants under the age of 18 were enrolled. Following a baseline study visit including consent and enrollment (Month 0), participants returned for 1- and 3-month follow-up visits. Each study visit included a urine pregnancy test, a venipuncture blood sample, an interviewer-administered survey through REDCap, and a cervicovaginal exam including the collection of cervicovaginal lavage (CVL) fluid and swabs for testing for STIs and bacterial vaginosis. Given the sensitive nature of the study, significant efforts were made to integrate trauma-informed practices into data collection procedures, which have been outlined in detail elsewhere [31]. Participants were compensated USD 50 for each study visit. All study procedures were approved by the University of California San Diego Institutional Review Board.

### 2.2. Interviewer-Administered Survey and Measures

Trained interviewers administered a 30–60 min REDCap survey at each study visit. The range of survey topics has been previously outlined [29]; for the present analysis, age (continuous), current use of hormonal birth control (yes/no), and mental health indicators were the only survey data employed. Mental health indicators included perceived stress (the Perceived Stress Scale, PSS), depressive symptoms (the Center for Epidemiologic Studies Depression Scale, CES-D-10), PTSD symptoms (the Primary Care PTSD Screener, PC-PTSD), and resilience (the Connor–Davidson Resilience Scale, CD-RISC). The PSS [32] contains ten items describing the negative experiences (e.g., being unable to control emotions) and emotions (e.g., feeling nervous or “stressed”) associated with stress. Case participants were asked to report the frequency they experienced each item during the past week on a five-point scale (1: “Never”, 5: “Very Often”) (Cronbach’s alpha = 0.97). The CES-D-10 [33] includes ten items describing different symptoms of depression (e.g., feeling lonely, restless sleep), with participants indicating the frequency they experienced each symptom in the past week on a four-point scale (1: “rarely or none of the time (<1 day),” 4: “most of the time (5–7 days)” (Cronbach’s alpha = 0.92). The PC-PTSD [34] contains four items detailing PTSD experiences such as feeling “constantly on guard, watchful, or easily startled.” Case participants endorsed yes/no to each experience with either the prompt, “since [their] recent experience with nonconsensual sex” (baseline) or “in the past month” (follow-up visits) (Cronbach’s alpha = 0.72). Finally, the CD-RISC [35,36] includes ten items regarding participants’ ability to cope with adversity (e.g., “I tend to bounce back after illness, injury, other hardships”) in the past week, each of which case participants rated on a five-point scale (0 = “not true at all,” 4: “true nearly all the time”) (Cronbach’s alpha = 0.89). For all mental health indicators, as appropriate, items were reverse-coded according to scoring instructions and then summed, resulting in continuous scales where higher values aligned with greater depression, PTSD symptomology, perceived stress, or resilience.

### 2.3. Biomarker Sample Collection and Processing

Venipuncture blood samples were collected at each visit by a trained phlebotomist and sent to the University of California San Diego lab for processing and testing of progesterone, in addition to other biomarkers not used in the current analysis [29]. Progesterone was quantified using the electrochemiluminescence immunoassay (ECLIA) (Roche Diagnostics, Indianapolis, IN, USA) in line with the manufacturer’s instructions; values were analyzed as a continuous variable. Additionally, blood samples were used for HIV-1/2 antibody and p24 antigen testing at each visit.

A trained clinician collected vaginal swabs that were sent for testing for gonorrhea, chlamydia, syphilis, trichomoniasis, and bacterial vaginosis (BV). CVL fluid was collected by rinsing the vaginal canal and cervix with 10 mL of normal saline (BD Biosciences, Franklin Lake, NJ, USA), which was allowed to pool in the posterior fornix and then collected by aspiration into a syringe. Samples were processed as described previously [29] and frozen at −80° until batch shipment on dry ice to the George Washington University for testing. Testing included pro-inflammatory cytokines/chemokines (interleukin [IL]-1α, IL-1β, IL-6, IL-8, TNF-α, and MIP-3 α) and anti-inflammatory/anti-HIV biomarkers (secretory leukocyte protease inhibitor (SLPI), elafin, and human β-defensin 2 (HBD2)), and was conducted using enzyme-linked immunosorbent assays (ELISA) from R&D Systems and PeproTech, with sensitivity of detection ≤ 1 pg/mL. Standards and all samples were run in triplicate; values < 15% coefficient of variation (CV) were determined to be acceptable. If values were undetectable, the lowest detectable value for a given biomarker assay was divided by half. The assay results were log-transformed using log base 10 and analyzed as continuous variables. A TZM-bl assay using the TZM-bl indicator cell-line described previously [37] was used to determine functional anti-HIV activity of CVL (HIV Reagent Program, NIH). A total of 10,000 cells per well were seeded in a 96-well plate and allowed to adhere overnight at 37 °C. Samples were diluted 1:1 in TZM-bl media (phenol red-free DMEM (Invitrogen Life Technologies, Carlsbad, CA, USA) supplemented with 10% defined FBS (HyClone, Logan, UT, USA)), 2 mM L-glutamine (Invitrogen Life Technologies), and 50 μg/mL Primocin (Invivogen, San Diego, CA, USA) for 2 h; then, laboratory-adapted CCR-5 tropic HIV BaL was added at multiplicity of infection (MOI) 0.5. Luciferase activity was measured after 48 h upon application of substrate beta-Glo (Promega, Madison, WI, USA). Uninfected cells and cells treated with CVL alone were used to determine background luminescence expressed as relative light units (RLUs). All conditions were tested in triplicate. Values < 15% CV were considered acceptable. To calculate percent inhibition, RLU values of “virus only” wells were averaged and set to 100%. Viability of cells upon treatment with CVL was quantified using the CellTiter 96^®^ AQueous One Solution Cell Proliferation Assay (Promega, Madison, WI, USA) according to the manufacturer’s instructions. Reagent was added directly to cell cultures and incubated for 30 min at 37 °C, followed by reading the plate in a plate reader at OD 490 nm. Values were used continuously in analyses. Prostate specific antigen (PSA) was tested as a marker for seminal fluid to rule out recent condomless vaginal sex and analyzed based on the manufacturer’s recommendations [38]. This is a standard approach, as the presence of seminal fluid can interfere with some of the biomarker assays.

### 2.4. Statistical Analysis

The current analysis used data from case participants only, at baseline (Month 0), follow-up one (Month 1), and follow-up two (Month 3). Using SPSS Version 26 [39], we calculated counts and percentages for categorical variables, means with standard deviations (SDs) for normally distributed continuous variables, and medians with interquartile ranges (IQRs) for non-normally distributed continuous variables. We employed paired *t*-tests to identify significant changes in mental health indicators and biomarkers between visits and used Pearson’s correlation coefficients to assess bivariate relationships between mental health and biomarkers at each timepoint. Using linear regressions, we created a series of adjusted and unadjusted models to understand the relationships between mental health indicators and biomarkers at each timepoint. Each mental health indicator (perceived stress, depression, PTSD, resilience) was used as the independent variable in a series of models. Within each series, Model A was unadjusted, Model B was adjusted for STI and BV diagnosis at the time of visit, age, progesterone as a marker of menstrual cycle stage, and current use of hormonal contraception, and Model C was adjusted for all covariates in Model B, plus any adverse mental health indicators that were not the predictor (e.g., perceived stress, depression, PTSD; not run with resilience). Model D included all covariates included in Model C, plus resilience. Regressions were estimated using Ordinary Least Squares (OLS). Missingness in the data was minimal. Significance was set at a level of *p* < 0.05, and two-sided *p*-values are reported where applicable. Given the log transformation of biomarkers, unstandardized regression coefficients are reported. Significance was set at *p* < 0.05.

## 3. Results

### 3.1. Participant Characteristics

Case participants (N = 25) had a median age of 24 (IQR:9) and were White (44.0%), Black (20%), Asian/Pacific Islander (20.0%), or Other (36.0%) (Table 1). Participants who self-categorized as “other” were primarily American Indian or Alaska Native (*n* = 2) or racially identified as Hispanic/Latina; of case participants, 13 (52.0%) identified ethnically as Hispanic/Latina. Over half of participants had at least some formal education beyond high school diploma or GED (52.0%) and were employed full- or part-time (60.0%).

### 3.2. Mental Health over Time

Participants self-reported perceived stress (PS), depressive symptoms, PTSD symptoms, and resilience via psychometric measures at each timepoint (Table 2). At baseline (BL), participants had a mean PS score of 18.72 (SD: 7.13, possible range: 0–40) and had a non-significantly higher mean PS score at Month 1 (M1: μ = 19.43, SD: 5.31). At Month 3 (M3), participants had a significantly lower PS score compared to both BL (μ = 18.72, SD: 7.13 vs. μ = 15.36, SD: 6.57, *p* = 0.041) and M1 (μ = 19.32, SD: 5.31 vs. μ = 15.36, SD: 6.57, *p* = 0.014). Mean depression score significantly decreased from BL to M1 (μ = 19.28, SD: 5.75 vs. μ = 14.20, SD: 5.78, *p* = 0.005), from M1 to M3 (μ = 14.20, SD: 5.78 vs. μ = 9.80, SD: 5.68, *p* = 0.003), and from BL to M3 (μ = 19.28, SD: 5.75 vs. μ = 9.80, SD: 5.68, *p* < 0.001). Mean PTSD symptom scores increased from BL to M1 (μ =2.76, SD: 1.59 vs. μ = 3.16, SD: 0.99), though not statistically significantly, and then significantly decreased from M1 to M3 (μ = 3.16, SD: 0.99 vs. μ = 2.27, SD: 1.35, *p* = 0.003). There were no statistically significant changes in mean resilience score across study visits (μ = 28.92, SD: 6.86 vs. μ = 27.52, SD: 5.79 vs. μ = 28.05, SD: 5.09).

### 3.3. Immune Biomarkers and HIV Inhibition over Time

Statistically significant changes in immune biomarkers over study visits were only identified for TNF-α and SLPI (Table 3). Compared to BL (μ = −1.41, SD: 1.45), mean TNF-α was significantly higher at M3 (μ = −0.39, SD: 1.91, *p* = 0.01) and marginally significantly higher at M1 (μ = −0.83, SD: 1.73, *p* = 0.091) (Supplemental Figure S1). There were no significant differences between M1 and M3. Compared to BL, the mean concentration of SLPI was significantly lower at M1 (μ = 5.65, SD: 1.13 vs. μ = 4.69, SD: 1.89, *p* = 0.048) (Supplemental Figure S2). There were no significant differences in SLPI concentration between BL and M3 or M1 and M3. There were no significant changes in percent HIV inhibition between visits.

### 3.4. Mental Health and Immune Biomarkers over Time

Correlations between each mental health indicator and each immune biomarker were assessed at each study timepoint (BL, M1, M3) (Table 4). PTSD symptomology was positively correlated with IL-1α at BL (r = 0.435, *p* = 0.038), and resilience was positively correlated with IL-1α at M3 (r = 0.498, *p* = 0.036); other than these individual relationships, PS, PTSD, and resilience were not statistically significantly correlated with any other biomarker at any timepoint. By contrast, depression was negatively correlated with TNF-α (r = −0.592, *p* = 0.003), IL-6 (r = −0.491, *p* = 0.017), and IL-1β (r = −0.417, *p* = 0.048) at BL, but not at other timepoints. Additionally, depression was negatively correlated with HBD2 at M3 (r = −0.604, *p* = 0.010).

Linear regressions results are presented in Table 5; biomarkers (e.g., elafin) and timepoints with no significant associations are not presented. PS was significantly positively associated with IL-8 at M1 in the minimally adjusted model (Model B, β = 0.216, 95% CI: 0.020, 0.413) and significantly negatively associated with HBD2 at M3 in the minimally adjusted model (Model B, β = −0.133, 95% CI: 0.259, −0.007). PS was also significantly associated with IL-1-α at BL in the model adjusting for all adverse mental health outcomes (Model C, β = −0.287, 95% CI: −0.500, −0.074) and all adverse mental health outcomes plus resilience (Model D, β = −0.343, 95% CI: −0.613, −0.073), with consistent directionality and a higher magnitude coefficient upon the inclusion of resilience.

Depression and PTSD symptomology demonstrated the most consistent association with immune biomarkers. At BL, depression was significantly associated across all models with TNF-α (β = −0.133–−0.152) and IL−6 (β = −0.171–−0.207), with consistent directionality. Additionally, depression was associated with IL-1-β in the unadjusted model (Model A: β = −0.181, 95%: (−0.360, −0.002). Together, these indicate lower inflammatory activity when more depressive symptoms were endorsed. While no significant associations between depression and examined biomarkers were identified at M1, depression was negatively associated with HBD2 at M3 in unadjusted (Model A, β = −0.176, 95% CI: −0.305, −0.048) and minimally adjusted models (Model B, β = −0.192, 95% CI: −0.347, −0.037), and depression was associated with lower percent HIV inhibition in the model accounting for other adverse mental health indicators and resilience (Model D, β = −3.962, 95% CI: 6.920, −0.944).

At BL, PTSD score was significantly positively associated with IL-1α across all models (β = 0.576–1.681), with a stepwise increase in the magnitude of the association in successive models. At M1, SLPI was consistently associated with PTSD in all regressions, except the unadjusted regression (Model A), indicating higher levels of SLPI as PTSD score increased (β = 1.974 to 2.081). PTSD was also positively associated with IL-6 at BL (β = 0.620, 95% CI: 0.073, 1.166) and with IL-8 at M1 (β = 1.719, 95% CI: 0.163, 3.274) in minimally adjusted models (Model B for each).

Finally, associations between resilience and biomarkers were only observed at M3. In the unadjusted regression (Model A), IL-1α was associated with resilience score; however, this association was not maintained in adjusted regressions. In the minimally adjusted model (Model B), resilience was associated with IL-6 (β = −0.235, 95% CI: −0.433, −0.037). In fully adjusted models (Model D for each), resilience was negatively associated with MIP-3α (β = −0363, 95% CI: −0636, −0.090) and percent HIV inhibition (β = −2.971, 95% CI: −4.818, −1.124).

## 4. Discussion

In this novel study of longitudinal follow-up of women who experienced recent rape, we observed changes in mental health indicators, HIV-associated biomarkers, and correlations between mental health indicators and biomarkers in the three months following sexual violence. Previous studies have explored mental health and immunity, including effects on mental health following trauma, but with variable findings [17,27,28]. Additionally, these studies typically consider peripheral immune biomarkers in blood/plasma samples and rarely investigate biomarkers in the female genital mucosa, the site of trauma in vaginal rape and the entry point for sexually transmitted pathogens including HIV. One recent study by Beattie et al. did assess this relationship, reporting no evidence of inflammation in the female genital tract (FGT) associated with violence exposure or poor mental health among Kenyan female sex workers [40], a group previously shown to exhibit a distinct genital immune profile [41,42]. In this study, women who experienced violence reported past six-month experience of either physical or sexual violence, indicating a longer timeline between violence and assessment of immunological response, as well as inclusion of women who may not have experienced forced vaginal sex [40]. The authors also assess inflammation specifically, as opposed to immune dysregulation. Combined with methodological differences known to influence FGT immune biomarker outcomes [43]—such as FGT sample collection technique (SoftCup versus cervicovaginal lavage), quantification platforms (Meso Scale Discovery versus ELISA), and analytic approaches (composite or binary inflammation measures versus individual biomarker analyses)—it is unsurprising that their findings contrast with our earlier work, where we observed alterations in genital immune biomarkers indicative of FGT immune dysregulation, and further noted that the nature of these alterations varied between recent and chronic (repeated) exposure to sexual violence [22,24]. For example, in a case study of a survivor of repeat victimization, we observed suppressed genital immune biomarkers correlating with increased adverse mental health, while mental health improvement correlated with immunological recovery [26]. In this case, increased resilience correlated with improved immune biomarkers regardless of revictimization and circumstantial changes [26]. The present analysis extends this work by identifying mental health recovery over the three months after sexual violence exposure and dynamic inflammatory and anti-inflammatory biomarker activity over time.

In bivariate analyses, we observed consistent, statistically significant decreases in depressive symptoms across all timepoints, while perceived stress and PTSD symptom scores significantly decreased by three-months post-rape compared to baseline. Interestingly, we did not see any changes in resilience scores over time, perhaps indicating high baseline resilience in this cohort, which was not impacted by recent exposure to sexual violence. Alternatively, our measure of resilience, the CD-RISC, is comprised of subscales that may be more general vs. specific to survivors or sexual violence (e.g., relational maintenance, independence, insight), resulting in reports of high resilience. This phenomenon was observed in another cross-sectional study on sexual violence using this scale [44].

We did not observe significant changes in most of the immune biomarkers over time, except for the pro-inflammatory biomarker TNF-α, which demonstrated a significant increase from BL to M3. Comparatively, the anti-inflammatory biomarker SLPI was significantly lower at M1 compared to baseline. TNF-α promotes inflammation and disrupts epithelial barriers, resulting in increased HIV acquisition, whereas SLPI has broad antimicrobial (including anti-HIV activity) and anti-inflammatory functions, particularly in the mucosa [45,46]. Although causality cannot be established in this case, it is possible that upregulation of SLPI occurs in response to TNF-α-mediated inflammation in the short-term following violence exposure, eventually resulting in resolution of the inflammatory response, as demonstrated by lower SLPI at M1 and M3 compared to BL. This hypothesis would align with findings of our recent comparative analysis of immune biomarkers among case and control participants at baseline in the same study [30], where we found that cases (recent non-consensual vaginal penetration) had significantly higher TNF-α and SLPI than controls (recent consensual vaginal penetration).

Despite the minimal significant changes identified among biomarkers across time, we did identify significant correlations between mental health indicators and biomarkers at different timepoints, along with corresponding associations in regression analyses. Depression was most frequently significantly negatively correlated with pro-inflammatory biomarkers, including with TNF-α, IL-6, and IL-1β at BL. While in adjusted regression analyses this relationship was not maintained with IL-1β, depression was significantly associated with both TNF-α and IL-6 across all adjusted regressions, providing evidence for a consistent relationship between these factors even when adjusting for STIs and endogenous (progesterone) and exogenous (hormonal birth control) hormones, as well as other mental health indicators. Depression frequently co-occurs with violence among women [17] and has been linked to HPA axis dysregulation and aberrant immune response [17]. For example, higher levels of peripheral IL-6 and IL-1β have previously been found in women with major depressive disorder compared to controls [47]. In the case of genital cytokines, we observed significant negative correlations, perhaps indicating an immune suppressive phenotype.

PTSD was moderately positively correlated with IL-1α at BL, a relationship which was retained across all regressions, with a higher magnitude regression coefficient upon successive adjustments. Importantly, depression is frequently comorbid with PTSD, and both have been associated with dysregulated immune responses and overall impaired health [48]. IL-1, including both IL-1α and IL-1β, bidirectionally impacts the HPA axis in addition to its role in immune regulation, which can result in neurobehavioral alterations in response to stress [49]. IL-1α is constitutively present in a variety of cells, including mucosal epithelial cells with barrier functions that are critical to immunological defense. IL-1α serves as an “alarm” in response to injury or damage [50], which activates IL-1β via an inflammasome-dependent mechanism. Previous studies have primarily examined IL-1β; therefore, a limited amount is known about the function of IL-1α relative to mental health. In the current study, more associations were identified between mental health indicators and IL-1α than IL-1β, particularly at the BL visit shortly after rape, which may be indicative of an ongoing and building activation of IL-1β in response to IL-1α that is not captured by the study timepoints. At M1, PTSD scores peaked and were positively associated with the anti-inflammatory/antimicrobial biomarker SLPI in all adjusted models. Since PTSD improves over time and SLPI can inhibit IL-1- and IL-6-associated inflammation [45], the loss of association with inflammatory IL-1α and IL-6 and gain of association with anti-inflammatory/antimicrobial SLPI at M1 may indicate a protective role of SLPI in the resolution of biological pathways that promote PTSD. SLPI also has direct and indirect HIV inhibitory functions; therefore, a gain of association in SLPI may indicate protective immune responses. At M3, no significant associations were observed between PTSD scores and any biomarkers, perhaps indicating behavioral and immunological resolution.

An interesting and novel finding in our study was regarding HBD2, which was moderately negatively correlated with depression at M3, a relationship maintained in minimally adjusted regressions, though not when other mental health indicators were controlled for. Additionally, while not reported, the *p*-value for the relationship between perceived stress and HBD2 at M3 was very near to the significance cutoff (*p* = 0.051). Defensins are a family of antimicrobial peptides that are expressed by multiple cell types in the body and can modulate immune responses by acting as alarmins to enhance immunity or suppressive to resolve immunity [51]. HBD2 is well characterized as a mucosal antimicrobial with anti-HIV functions [52,53]. HBD2 is also a chemokine that binds to the receptor CCR6, enabling the recruitment of T cells and dendritic cells to the site of microbial invasion or injury [52]. Although data on associations between mental health indicators is sparse, a small but growing literature suggests biological plausibility, where changes in HBD2 could reflect dysregulation of the gut–brain axis and affect neuroinflammation. A study of pregnant people found that high perceived stress and low cervicovaginal HBD-2 were associated with increased odds of spontaneous preterm birth—an example of psychosocial stress mapping onto local defensin levels and downstream health outcomes [54]. Emerging psychiatric research also suggests associations between certain psychotropic medications and peripheral beta-defensin-2 levels, although there are a few direct psychiatric case–control studies measuring HBD-2 in diagnosed depression, anxiety, PTSD, psychosis. In our previous studies in women exposed to chronic sexual abuse, we found HBD2 to be at significantly lower levels in plasma but not in the genital tract in those reporting chronic abuse and current depression compared to controls without depression and abuse exposure [23]. The finding that, at M3, decreased perceived stress and depression were associated with increased HBD2 suggests a previously uncharacterized role of HBD2, as a protective immune modulator. This is important, as depression is consistently associated with higher risk of HIV acquisition, driven by behavioral and biological immune dysregulation.

Another interesting observation was the significant negative association between resilience and MIP-3α at M3, after controlling for biological and mental health indicators. MIP-3α functions as both a homeostatic and inflammatory chemokine through interactions with T cells and dendritic cells [55,56], shares its receptor with HBD2, and functions as an antimicrobial with anti-HIV activity [55,56]. Therefore, the role of MIP-3α may be protective through its antimicrobial activity or by recruiting immune cells to the site of pathogen entry. However, as the MIP-3α-recruited immune cells are also targets for HIV infection, this may be a double-edged sword. Resilience was also similarly associated with percent HIV inhibition, a functional measurement based on the combined effects of the extent to which multiple biologically active immune biomarkers in genital secretions inhibit HIV in vitro [37]. In previous studies, we identified positive correlations between FGT secretion anti-HIV activity and both MIP-3α and HBD2 [37]. Resilience is impacted by immune processes, and both human and animal studies have shown that it is possible to render stress-susceptible individuals resilient, and vice versa, by changing their inflammatory phenotype in experimental models [57]. Although resilience scores did not change in our participants during the three-month follow-up, our data indicates subtle interactions between immune biomarkers and resilience, which were perhaps not captured by the measurement methodology.

Despite its novel, exploratory nature, there are limitations to be considered in interpretation of the findings. While a variety of recruitment methods were employed for this study, the relatively high burden of interview completion and biomarker sample collection may bias the sample toward women who are more motivated to and comfortable with research participation. For this and other reasons, our study may not be generalizable, despite it being relatively racially and ethnically diverse. Mental health indicators are based on self-report, though all measures employed in the study were validated and have adequate consistency, as indicated by Cronbach’s alpha. However, as noted, previous studies with violence-exposed populations have noted potential ceiling effects in the measure of resilience used [44]. Temporality and origin of mental health cannot be determined; mental health at the time of each is likely the result of myriad exposures, possibly pre-dating exposure to sexual violence. It is not possible to attribute variance in these indicators to sexual violence vs. non-sexual violence experiences; therefore, any associations may be representative of causal pathways external to sexual violence exposure. Regressions were run using cross-sections of the data, limiting any causal interpretation. However, given the time between data collection points and the relatively rapid changes in biomarker concentrations possible, prospective modeling of M1 or M2 biomarker concentrations as a function of BL or M1 mental health status seems unlikely to yield informative and actional insights. More frequent sampling may be an area of further development and research. The sample size for this analysis is small, and several factors are adjusted due to the complexity of the relationships being examined and the limited likelihood of bivariate analyses meaningfully indicating associations within such relationships. This limits statistical power and increases the likelihood of spurious results. Relationships that are maintained across regression models are given more consideration for this reason. Regression analyses do not account for the possibility of interactive effects between mental health indicators, which may warrant examination in studies with sufficient sample sizes.

Regardless of these limitations, our study provides novel evidence linking adverse mental health indicators with genital immune biomarkers associated with HIV acquisition risk. Specifically, our findings suggest that anti-HIV biomarkers, including SLPI and HBD2, may play a protective role by mitigating adverse mental health outcomes and reducing HIV susceptibility over time. Moreover, the data indicate that post-rape inflammatory activity within the FGT is associated with symptoms of depression and PTSD, even after adjusting for potential confounders. Leveraging our unique longitudinal post-rape cohort, these findings offer new opportunities to enhance the health and well-being of sexual violence survivors and highlight important directions for future research regarding HIV prevention.

## Figures and Tables

**Table 1:** Study participant characteristics, San Diego, CA, 2018 to 2020 (N = 25).

Variables	N (%) or Median (IQR)
**Age** (years)	24 (9)
**Race** (Not Mutually Exclusive)	
Black/African American	5 (20.0)
White	11 (44.0)
Asian/Pacific Islander	5 (20.0)
Other	9 (36.0)
**Ethnicity**	
Hispanic/Latinx	13 (52.0)
**Educational Attainment**	
High School Diploma, GED, or Less	12 (48.0)
Some Trade, Vocational School, or College or more	13 (52.0)
**Employment Status**	
Unemployed	10 (40.0)
Employed Part-Time	4 (16.0)
Employed Full-Time	11 (44.0)

**Table 2:** Mental health indicators among recent survivors of sexual violence at baseline, 1 month, and 3 months (N = 25).

Mental Health Indicator	Baseline	1 Month	3 Months	*p*		
				BL vs. M1	M1 vs. M3	BL vs. M3
Perceived Stress	18.72 (7.13)	19.32 (5.31)	15.36 (6.57)	0.715	**0.014**	**0.041**
Depression	19.28 (5.75)	14.20 (5.78)	9.80 (5.68)	**0.005**	**0.003**	**<0.001**
PTSD	2.76 (1.59)	3.16 (0.99)	2.27 (1.35)	0.317	**0.003**	0.388
Resilience	28.92 (6.86)	27.52 (5.79)	28.05 (5.09)	0.350	0.348	0.655

Bolded values indicate significance at a level of <0.05. BL, baseline; M1, Month 1; M3, Month 3.

**Table 3:** Log-normalized biomarkers among recent survivors of sexual violence at baseline, 1 month, and 3 months (N = 25).

Biomarker	Baseline	1 Month	3 Months	*p*		
				BL vs. M1	M1 vs. M3	BL vs. M3
IL-8	1.85 (1.47)	1.16 (1.88)	2.21 (1.93)	0.164	0.083	0.544
MIP-3α	4.38 (2.47)	3.59 (2.85)	4.89 (1.76)	0.164	0.086	0.523
TNF-α	−1.41 (1.45)	−0.83 (1.73)	−0.39 (1.91)	0.091	0.111	**0.010**
IL-1 α	−0.24 (1.82)	−0.47 (1.78)	0.18 (0.99)	0.605	0.237	0.556
IL-6	1.55 (2.34)	1.39 (2.26)	2.51 (2.32)	0.798	0.172	0.259
IL-1β	−1.41 (2.48)	−1.33 (2.01)	−0.41 (2.67)	0.861	0.263	0.372
Elafin	6.57 (1.10)	6.58 (1.25)	7.13 (0.87)	0.949	0.196	0.132
HBD2	2.17 (1.47)	1.84 (1.46)	2.70 (1.87)	0.310	0.076	0.360
SLPI	5.65 (1.13)	4.69 (1.89)	5.00 (1.57)	**0.048**	0.432	0.412
% HIV Inhibition	30.9 (21.74)	31.67 (23.00)	23.86 (21.06)	0.911	0.214	0.210

Bolded values indicate significance at a level of <0.05. BL, baseline; M1, Month 1; M3, Month 3.

**Table 4:** Correlation coefficients for log-normalized biomarkers with mental health indicators among recent survivors of sexual violence at baseline, 1 month, and 3 months (N = 25).

	Perceived Stress			Depression			PTSD			Resilience		
Biomarker	BL	M1	M3	BL	M1	M3	BL	M1	M3	BL	M1	M3
IL-8	0.026	0.409	−0.291	−0.095	0.025	−0.314	0.130	0.204	−0.059	−0.173	−0.104	0.051
MIP-3α	0.072	0.230	−0.106	−0.061	−0.070	−0.071	−0.108	−0.165	−0.241	−0.234	−0.181	−0.366
TNF-α	−0.059	0.062	0.151	**−0.592**	0.121	0.129	−0.001	0.234	−0.079	0.105	−0.104	−0.274
IL-1α	−0.014	0.155	−0.304	−0.217	0.167	−0.325	**0.435**	0.053	0.012	0.175	0.263	**0.498**
IL-6	0.169	0.011	−0.15	**−0.491**	−0.157	0.018	0.207	−0.289	−0.124	−0.024	0.116	−0.194
IL-1β	−0.377	0.073	−0.354	**−0.417**	−0.092	−0.480	−0.084	−0.314	−0.085	0.138	−0.150	0.266
Elafin	0.002	0.107	−0.120	−0.045	0.322	−0.195	−0.019	0.139	0.025	−0.041	−0.197	0.378
HBD2	−0.029	0.110	−0.461	−0.027	0.076	**−0.604**	−0.049	0.154	−0.242	0.190	−0.154	0.378
SLPI	−0.110	0.358	−0.341	−0.105	0.114	−0.234	−0.017	0.404	−0.431	−0.023	−0.298	0.114
% HIV Inhibition	−0.066	0.310	−0.134	0.072	0.360	−0.189	−0.200	0.285	−0.037	0.032	−0.328	−0.187

Bolded values indicate significance at a level of <0.05. BL, baseline; M1, Month 1; M3, Month 3.

**Table 5:** Linear regression coefficients for log-normalized biomarkers with mental health indicators across timepoints among recent survivors of sexual violence (N = 25).

Perceived Stress									
Timepoint	Model	IL-8	IL-1α						HBD2
Baseline	A	0.005 (−0.085, 0.96)	−0.004 (−0.149, 0.140)						−0.006 (−0.095, 0.084)
	B	0.065 (−0.034, 0.164)	0.061 (−0.127, 0.250)						0.035 (−0.084, 0.154)
	C	0.029 (−0.148, 0.207)	**−0.287 (−0.500, −0.074)**						0.094 (−0.113, 0.302)
	D	−0.024 (−0.247, 0.198)	**−0.343 (−0.613, −0.073)**						0.157 (−0.104, 0.418)
Month 1	A	0.157 (−0.022, 0.336)	0.055 (−0.126, 0.237)						0.032 (−0.116, 0.181)
	B	**0.216 (0.020, 0.413)**	0.164 (−0.041, 0.370)						0.016 (−0.184, 0.215)
	C	0.112 (−0.151, 0.376)	0.083 (−0.226, 0.391)						−0.088 (−0.376, 0.200)
	D	0.106 (−0.198, 0.410)	0.175 (−0.111, 0.461)						−0.087 (−0.420, 0.245)
Month 3	A	−0.075 (−0.206, 0.056)	−0.052 (−0.138, 0.034)						−0.107 (−0.217, 0.002)
	B	−0.038 (−0.202, 0.127)	−0.019 (−0.134, 0.097)						**−0.133 (−0.259, −0.007)**
	C	−0.038 (−0.356, 0.280)	0.009 (−0.188, 0.205)						−0.044 (−0.268, 0.181)
	D	−0.024 (−0.353, 0.305)	−0.007 (−0.177, 0.163)						−0.044 (−0.292, 0.203)
**Depression**									
**Timepoint**	**Model**	**TNF-α**	**IL-6**		**IL-1β**		**HBD2**		**% HIV Inhibition**
Baseline	A	**−0.133 (−0.215, −0.051)**	**−0.207 (−0.372, −0.040)**		**−0.181 (−0.360, −0.002)**		−0.006 (−0.111, 0.099)		0.266 (−1.396, 1.928)
	B	**−0.141 (−0.247, −0.035)**	**−0.184 (−0.347, −0.021)**		−0.140 (−0.309, 0.030)		0.041 (−0.087, 0.170)		0.076 (−2.070, 2.223)
	C	**−0.149 (−0.269, −0.030)**	**−0.171 (−0.319, −0.023)**		−0.106 (−0.286, 0.073)		0.022 (−0.118, 0.163)		−0.020 (−2.421, 2.381)
	D	**−0.152 (−0.271, −0.032)**	**−0.171 (−0.326, −0.016)**		−0.105 (−0.293, 0.083)		0.025 (−0.119, 0.168)		−0.022 (−2.544, 2.500)
Month 3	A	0.046 (−0.148, 0.239)	0.007 (−0.203, 0.127)		−0.211 (−0.423, 0.001)		**−0.176 (−0.305, −0.048)**		−0.771 (−2.971, 1.428)
	B	0.119 (−0.147, 0.386)	0.226 (−0.024, 0.477)		−0.095 (−0.348, 0.158)		**−0.192 (−0.347, −0.037)**		−0.418 (−3.045, 2.208)
	C	0.226 (−0.192, 0.634)	0.300 (−0.131, 0.732)		−0.256 (−0.695, 0.183)		−0.171 (−0.454, 0.112)		−2.127 (−6.592, 2.337)
	D	0.142 (−0.311, 0.595)	0.175 (−0.241, 0.592)		−0.306 (−0.813, 0.200)		−0.167 (−0.502, 0.168)		**−3.962 (−6.920, −0.944)**
**PTSD**									
**Timepoint**	**Model**	**IL-8**	**IL-1α**			**IL-6**			**SLPI**
Baseline	A	0.108 (−0.267, 0.482)	**0.576 (0.035, 1.116)**			0.309 (−0.354, 0.971)			−0.012 (−0.342, 0.318)
	B	0.253 (−0.103, 0.609)	**0.774 (0.226, 1.323)**			**0.620 (0.073, 1.166)**			0.060 (−0.287, 0.408)
	C	0.170 (−0.466, 0.805)	**1.591 (0.825, 2.356)**			0.683 (−0.099, 1.466)			0.031 (−0.588, 0.650)
	D	0.256 (−0.423, 0.935)	**1.681 (0.856, 2.506)**			0.671 (−0.192, 1.534)			0.053 (−0.628, 0.735)
Month 1	A	0.381 (−0.555, 1.318)	0.093 (−0.800, 0.985)			−0.667 (−1.797, 0.464)			0.743 (−0.118, 1.604)
	B	**1.719 (0.163, 3.274)**	1.263 (−0.380, 2.906)			−0.078 (−2.202, 2.045)			**2.081 (0.618, 3.543)**
	C	1.343 (−0.166, 2.851)	0.974 (−0.791, 2.739)			−0.288 (−2.703, 2.127)			**2.057 (0.336, 3.779)**
	D	1.320 (−0.349, 2.989)	1.285 (−0.238, 2.854)			−0.103 (−2.687, 2.481)			**1.974 (0.093, 3.856)**
**Resilience**									
**Timepoint**	**Model**	**MIP-3α**	**IL-1α**			**IL-6**			**% HIV Inhibition**
Month 3	A	−0.139 (−0.326, 0.048)	**0.109 (0.008, 0.209)**			−0.075 (−0.276, 0.126)			−0.808 (−3.060, 1.444)
	B	−0.245 (−0.429, 0.001)	0.105 (−0.008, 0.218)			**−0.235 (−0.433, −0.037)**			−2.166 (−4.399, 0.067)
	D	**−0.363 (−0.636, −0.090)**	0.124 (−0.017, 0.265)			−0.202 (−0.457, 0.053)			**−2.971 (−4.818, −1.124)**

Bolded values indicate significance at a level of <0.05. Model A is unadjusted. Model B is adjusted for STI (none/any), bacterial vaginosis (yes/no), age, progesterone (ng/mL), and hormonal birth control use (non/any, includes copper IUD). Model C is adjusted for STI (none/any), bacterial vaginosis (yes/no), age, progesterone (ng/mL), hormonal birth control use (non/any, includes copper IUD), and non-outcome adverse mental health indicators (perceived stress, depression, PTSD). Model D is adjusted for STI (none/any), bacterial vaginosis (yes/no), age, progesterone (n)/mL, hormonal birth control use (non/any, includes copper IUD), non-outcome adverse mental health indicators (perceived stress, depression, PTSD), and resilience (adverse mental health predictors only). Within each mental health indicator, only biomarkers with a significant coefficient at ≥1 timepoint are reported, and only timepoints with at least one significant biomarker coefficient are reported (e.g., for perceived stress, only IL-8, IL-1α, and HBD2 were significantly associated with perceived stress at any timepoint; for depression, no biomarkers were significantly associated with depression at Month 1).

## Data Availability

Data is available upon reasonable request to the corresponding author.

## Supplementary Materials

The following supporting information can be downloaded at https://www.mdpi.com/article/10.3390/v18010119/s1. Figure S1: Log Normalized Concentration of TNF-α among Recent Survivors of Sexual Violence at Baseline, 1 Month, and 3 Months; Figure S2: Log Normalized Concentration of SLPI among Recent Survivors of Sexual Violence at Baseline, 1 Month, and 3 Month.

## Abbreviations

The following abbreviations are used in this manuscript:

AIDS	Acquired Immunodeficiency Syndrome
BL	Baseline
CV	Coefficient of Variation
CD-RISC	Connor–Davidson Resilience Scale
CES-D-10	Center for Epidemiologic Studies Depression Scale
CRP	C-Reactive Protein
CVL	Cervicovaginal Lavage
DMEM	Dulbecco’s Modified Eagle Medium
ECLIA	Electrochemiluminescence Immunoassay
ELISA	Enzyme-linked Immunosorbent Assay
FBS	Fetal Bovine Serum
FGT	Female Genital Tract
GED	General Educational Development
HIV	Human Immunodeficiency Virus
HPA	Hypothalamic–Pituitary–Adrenal
HBD2	Human β-defensin 2
IL-1α	Interleukin-1α
IL-1β	Interleukin-1β
IL-2	Interleukin-2
IL-6	Interleukin-6
IL-8	Interleukin-8
IL-10	Interleukin-10
IFN-γ	Interferon-γ
IP-10	Inducible Protein-10
IPV	Intimate Partner Violence
IQR	Interquartile Range
M1	Month 1
M3	Month 3
MCP-1	Monocyte Chemoattractant Protein-1
MIP-1α	Macrophage Inflammatory Protein-1α
MIP-1β	Macrophage Inflammatory Protein-1β
MIP-3α	Macrophage Inflammatory Protein-3α
PS	Perceived Stress
PSA	Prostate Specific Antigen
PSS	Perceived Stress Scale
PC-PTSD	Primary Care PTSD Screener
PTSD	Post-Traumatic Stress Disorder
REDCap	Research Electronic Data Capture
RLU	Relative Light Units
SD	Standard Deviation
SLPI	Secretory Leukocyte Protease Inhibitor
SPSS	Statistical Package for the Social Sciences
STI	Sexually Transmitted Infection
TNF-α	Tumor Necrosis Factor-α
