# Supplementary figures and images for: Mental Health, Mucosal Immunity, and HIV Susceptibility Following Sexual Violence: Evidence from the THRIVE Study
Source: Viruses. 2026 Jan 15;18(1):119. doi: 10.3390/v18010119 (PMC12846484; doi:10.3390/v18010119)

Supplemental Figure S1.

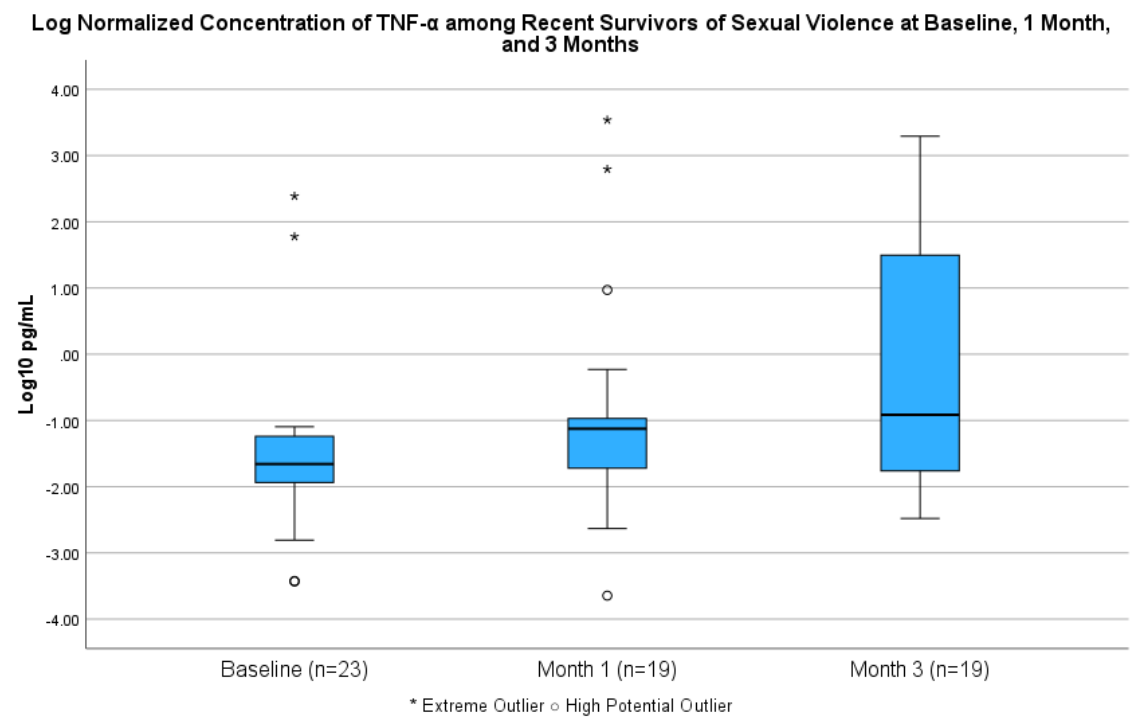

Supplemental Figure S2.

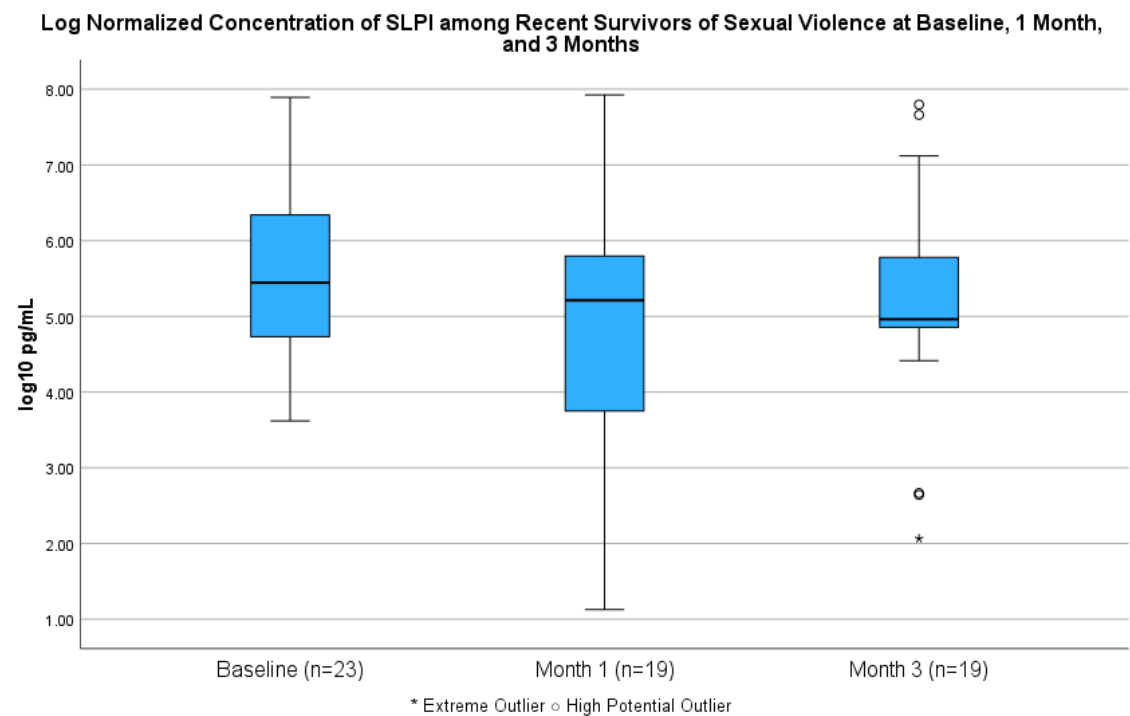

Supplement: Supplementary file 1 [file viruses-18-00119-s001.zip › viruses-4026486-supplementary.pdf]
